# Revolutionizing neurosurgical care: Cost-effective follow-up with social media applications

**DOI:** 10.12669/pjms.40.12(PINS).11104

**Published:** 2024-12

**Authors:** Raana Shahid, Zainab Safdar

**Affiliations:** 1 Dr. Raana Shahid, MBBS. Department of Neurosurgery, Punjab Institute of Neurosciences Sciences, Lahore, Pakistan; 2 Dr. Zainab Safdar, MBBS. Department of General Surgery, Lahore General Hospital, Lahore, Pakistan

Pakistan, a nation long recognized as a developing country by the United Nations Organization (UNO), faces significant challenges in its healthcare landscape. Despite boasting 22 College of Physicians and Surgeons (CPSP) accredited institutions and 177 registered neurosurgery residents in training, the disparity between available resources and demand remains glaring. With only 212 trained neurosurgeons nationwide, averaging just one neurosurgeon for every million citizens, Pakistan lags far behind global standards. This deficiency is acutely felt in cases of emergency trauma, particularly those arising from road traffic accidents, which contribute substantially to the country’s neurosurgical caseload. Compounded by the unequal distribution of resources, most critical cases are funneled towards major cities, exacerbating the burden on already strained facilities.^1^

While it is evident that both operated and non-operated cases require proper and long-term follow-up, particularly trauma cases such as extradural hematomas, subdural hematomas, and bone fractures, there is often a failure to ensure such monitoring. This is crucial to assess recovery and watch for potential complications such as re-bleeds, cerebrospinal (CSF) leaks, or other adverse effects. Moreover, cases requiring extended follow-ups, such as spine and brain tumor cases, frequently slip through the cracks due to several factors contributing to patients being lost to follow-up, which can be categorized into immediate and distal variables. Immediate factors pertain to the healthcare system itself, while distal factors include individual patient characteristics as well as sociological, cultural, and economic aspects.^2^ Compounding this issue is the fact that significant proportion of patients are living far from the treating institutions, particularly in remote northern cities, where repeated visits are logistically challenging. Additionally, a considerable number of patients come to the tertiary care hospitals in big cities, like Lahore from neighboring countries like Iran and Afghanistan, further complicating the follow-up process.

Social media applications like WhatsApp, Viber and Skype offer valuable opportunities for patient follow-up in healthcare settings, providing instant communication channels that enable healthcare providers to maintain regular contact with patients and address concerns promptly. These platforms are cost-effective alternatives, reducing the need for physical appointments and travel expenses of patients. Remote monitoring capability allow providers to track patients’ progress, medication adherence, and symptoms, particularly beneficial for those in far flung areas. Additionally, these apps support various forms of communication, including text, voice, and video calls, facilitating comprehensive assessments of patients’ conditions. Appointment reminders and medication schedules can be easily sent, ensuring patients stay on track with their treatment plans.^2^

Moreover, social media platforms serve as a means to share educational resources, empowering patients to better understand their conditions and treatment options. This system bridges the gap between periphery and center, streamlining patient care and reducing the burden on outpatient clinics. By centralizing patient data, it addresses issues with medical record management and reduces the risk of fragmented care due to doctor shopping. Hoz et al. conducted a study in Iraq in 2022 in which the utilized WhatsApp as a communication tool for over 200 post operative cerebrovascular cases. WhatsApp proved as a cost-effective application with increased compliance rates for the recruited patients.^2^ Another study conducted by Pencle et al. in 2018 revealed that 92% of patients in strong favor of utilization of mobile video conferencing (VC) for communicating with the surgeon, particularly for discussing the surgical procedure and postoperative care. Additionally, 83% of patients perceived that having access to a mobile video app suggested that their surgeon was more caring preoperatively, compared to 89% who felt the same postoperatively.^3^

In a country with low patient education levels, telemedicine via WhatsApp enables effective communication and instruction between patients and providers, enhancing overall healthcare delivery and outcomes. WhatsApp’s secure platform, bolstered by end-to-end encryption, ensures patients’ privacy is safeguarded during communication. Its real-time cloud communication capabilities make it an optimal platform for healthcare providers to promptly address patient inquiries, thereby enhancing overall patient experiences. The ability to easily save, search, and access WhatsApp messages facilitates the creation of a comprehensive record of patients’ medical history, simplifying communication tracking and immediate response to inquiries. Given its widespread usage and user-friendly interface, WhatsApp is highly valued by patients and serves as a cost-effective marketing tool for healthcare providers. While physical examinations aren’t possible via social media, providers can still assess mobility and wound status. Although it doesn’t replace in-person visits, some follow-up is better than none.

Integrating WhatsApp as a follow-up tool in healthcare practices offers a multitude of benefits, but its effective utilization requires caution. Firstly, establishing individual WhatsApp account for each healthcare provider ensures professionalism and privacy in communication. Secondly, scheduling follow-up video call on specified day and time facilitates structured interactions, enhancing patient-provider communication. Plan should be to implement WhatsApp as a follow-up tool, healthcare facilities should first collect each patient’s contact information, the social media applications they use, and the contact numbers of at least three immediate family members upon discharge. Surgeons and physicians should ensure they have access to all the apps their patients use, such as WhatsApp, Facebook Messenger, Skype, IMO etc. to facilitate online follow-ups. Clear instructions should be provided to patients regarding call preparation and technical usage of WhatsApp. Maintaining comprehensive documentation of all interactions is crucial for record-keeping and continuity of care. Prioritizing patient privacy and confidentiality by avoiding group chats for sensitive discussions is essential. These practices can optimize WhatsApp’s potential as a valuable tool for patient follow-up, fostering improved communication, convenience, and patient satisfaction in healthcare delivery.

## Authors Contribution

**RS** conceived, designed the study and drafted manuscript.

**ZS** did literature search and critical revision.

Both authors have approved the final version and are accountable for the work of submission.
